# A Mobile and Web-Based Self-Directed Complementary and Integrative Health Program for Veterans and Their Partners (Mission Reconnect): Protocol for a Mixed-Methods Randomized Controlled Trial

**DOI:** 10.2196/13666

**Published:** 2019-05-13

**Authors:** Jolie N Haun, Lisa M Ballistrea, Christine Melillo, Maisha Standifer, Kevin Kip, Jacquelyn Paykel, Jennifer L Murphy, Carol E Fletcher, Allison Mitchinson, Leila Kozak, Stephanie L Taylor, Shirley M Glynn, Matthew Bair

**Affiliations:** 1 Rehabilitation Outcomes Research Section James A Haley Veterans’ Hospital and Clinics Veterans Health Administration Tampa, FL United States; 2 College of Public Health University of South Florida Tampa, FL United States; 3 Whole Health Service James A Haley Veterans’ Hospital and Clinics Veterans Health Administration Tampa, FL United States; 4 Mental Health and Behavioral Sciences Service James A Haley Veterans’ Hospital and Clinics Veterans Health Administration Tampa, FL United States; 5 Veterans Affairs Ann Arbor Healthcare System Veterans Health Administration Ann Arbor, MI United States; 6 Department of Family Medicine University of Washington School of Medicine University of Washington Seattle, WA United States; 7 Veterans Affairs Puget Sound Health Care System Veterans Health Administration Seattle, WA United States; 8 Integrative Health Coordinating Center Office of Patient Centered Care and Cultural Transformation Veterans Health Administration Washington, DC United States; 9 Health Services Research and Development Veterans Health Administration Los Angeles, CA United States; 10 Department of Health Policy and Research University of California - Los Angeles Los Angeles, CA United States; 11 Research Service Veterans Affairs Greater Los Angeles Healthcare System at West Los Angeles Los Angeles, CA United States; 12 School of Medicine Indiana University Indianapolis, IN United States; 13 Regenstrief Institute, Inc Indianapolis, IN United States; 14 Center for Health Information and Communication Veterans Affairs Indianapolis, IN United States

**Keywords:** health information technology, implementation, veteran, complementary and integrative health, PTSD, pain

## Abstract

**Background:**

Complementary and integrative health (CIH) is a viable solution to PTSD and chronic pain. Many veterans believe CIH can be performed only by licensed professionals in a health care setting. Health information technology can bring effective CIH to veterans and their partners.

**Objective:**

This paper describes the rationale, design, and methods of the Mission Reconnect protocol to deliver mobile and Web-based complementary and integrative health programs to veterans and their partners (eg, spouse, significant other, caregiver, or family member).

**Methods:**

This three-site, 4-year mixed-methods randomized controlled trial uses a wait-list control to determine the effects of mobile and Web-based CIH programs for veterans and their partners, or dyads. The study will use two arms (ie, treatment intervention arm and wait-list control arm) in a clinical sample of veterans with comorbid pain and posttraumatic stress disorder, and their partners. The study will evaluate the effectiveness and perceived value of the Mission Reconnect program in relation to physical and psychological symptoms, global health, and social outcomes.

**Results:**

Funding for the study began in November 2018, and we are currently in the process of recruitment screening and data randomization for the study. Primary data collection will begin in May 2019 and continue through May 2021. Projected participants per site will be 76 partners/dyads, for a total of 456 study participants. Anticipated study results will be published in November 2022.

**Conclusions:**

This work highlights innovative delivery of CIH to veterans and their partners for treatment of posttraumatic stress disorder and chronic pain.

**Trial Registration:**

ClinicalTrials.gov NCT03593772; https://clinicaltrials.gov/ct2/show/NCT03593772 (Archived by WebCite at http://www.webcitation.org/77Q2giwtw)

**International Registered Report Identifier (IRRID):**

PRR1-10.2196/13666

## Introduction

### Background

Chronic pain and posttraumatic stress disorder (PTSD) are highly prevalent comorbid conditions in the veteran population and present a challenge for traditional interventions [1,2]. Pharmacological options have potential consequences of opioid use disorder and overdose [3]. The Veterans Affairs (VA)’s Opioid Safety Initiative and the US Department of Health and Human Services 2016 National Pain Strategy prioritize the need for nonpharmacological pain treatment options that leverage self-management [4,5].The VA is invested in providing virtual services to improve veteran and family member access to care that promotes self- management and improves patient outcomes. The Veterans Health Administration (VHA) currently views touch-based therapies as interventions requiring professional delivery. Massage costs approximately US $60/hour [6], is primarily an out-of-pocket expense, and is often not affordable. Massage is not currently widely available in the VA; however, with the emerging Whole Health initiative, massage and other mind-body modalities will be in increasing demand. Simply put, the VA will not be able to meet the increasing demand for massage services.

Mission Reconnect (MR) provides a potentially low-cost, accessible, and sustainable intervention for veterans in home settings. The need for low-cost interventions to support well-being and optimal functioning among veterans and their families is clear [7]. Furthermore, the VHA’s Secretary for Health’s Critical Priorities for Strategic Action identified access, pain management, and putting veterans first to achieve health for veterans. To be responsive to these priorities, the 2016 VA State-of-the-Art Conference and VA Comprehensive Addiction and Recovery Act mandate [8] indicate VA’s commitment to conduct rigorous research to integrate nonpharmacological and complementary and integrative health (CIH) approaches into care at every level, with emphasis on pain management. To support these priorities, the Office of Patient-Centered Care and Cultural Transformations’ Whole Health Program provides an innovative approach to integrative care that leverages CIH and family participation. Finally, the Connected Care Office seeks to support self-care management and health care using mobile technology.

Severity of outcomes, cost of care, and initiatives prioritizing access to CIH and whole health care warrant the need for a multidimensional approach, such as MR. The proposed research will advance science by (1) testing a safe self-directed sustainable CIH approach for treating a clinically defined high-risk population of veterans diagnosed with chronic pain and PTSD-related symptoms to improve psychological and physical outcomes, (2) introducing a nonpharmacologic chronic pain management program option for veterans, (3) testing the usefulness of MR as a remotely delivered Internet/mobile program delivered in the users’ natural environment, (4) leveraging a partnered approach (eg, spouses or family members) to implementing interventions that address the biopsychosocial aspect of wellness, and (5) conducting longitudinal repeated-measures analyses, which are not common in CIH research.

### Impact of Chronic Pain

Chronic pain is a highly prevalent condition among veterans (81.5%) [1]. Musculoskeletal ailments are some of the most frequent reasons that veterans seek care at the VA [2]. In addition to discomfort and mood and sleep disturbances associated with pain, veterans with chronic pain have a high coprevalence of medical, mental health, and substance use disorders [4]. Veterans with pain have higher prescribed opioid doses, which is associated with risk of accidental poisoning death and suicide death [6]. In sum, chronic pain is a high priority area in the VA due to its prevalence and severity of impact on quality of life for veterans and their families.

### Compounded Impact of Chronic Pain and Comorbid Posttraumatic Stress Disorder on Outcomes

We chose to address chronic pain and PTSD due to their prevalence, compounded impact, and their priority in the VA. As many as 70% of veterans with chronic pain treated in the VA have PTSD, and up to 80% of those with PTSD have pain [2]. Prevalence of PTSD is higher in patients with chronic pain [8,9]. Patients with both PTSD and chronic pain generally present with more complicated clinical profiles [10], and patients with these comorbidities report lower quality of life than their veteran counterparts [11]. Chronic pain and PTSD are associated with high rates of depression, anxiety, and fatigue [12-16], which detrimentally impact work, social functioning, relationships, independent living, and ability to enjoy life [17-19]. Effectiveness of pharmacotherapy is limited and can result in other negative consequences, such as substance use disorders [20]. Further, veterans with PTSD receive more frequent and higher-dose opioids for pain diagnoses than veterans without PTSD [6]. Innovative CIH approaches are needed to help veterans and their families cope with chronic pain and PTSD without the side effects and adverse events associated with pharmacological management [21-23].

### Theoretical Mechanisms Connecting Pain and Posttraumatic Stress Disorder

The biopsychosocial model represents the complex interrelationships between physical, psychological, and social factors [24,25]. Within the context of our research, the application of the biopsychosocial model will center around the bidirectional relationship between pain and PTSD. Psychological trauma induces change in biological substrates, which in turn alter pain transduction pathways and pain processing mechanisms in the brain, intensifying pain experience in individuals with PTSD [2]. Neurologically, PTSD is characterized by hyperactivation of the amygdala and hippocampus, and lower activation and imbalance in the medial prefrontal cortex. The amygdala integrates nociceptive information and plays a dual faciliatory and inhibitory role in the modulation of emotional pain behavior [2]. Therefore, interventions that ameliorate pain may be expected to reduce symptoms of PTSD [26] and vice versa [2]. On the basis of this bidirectional biopsychosocial model, we propose a multidimensional CIH intervention to support positive multifactorial outcomes associated with comorbid chronic pain and PTSD.

Veterans’ chronic conditions can affect their families’ well-being. In alignment with the biopsychosocial model, we contend the complexity of comorbid chronic pain and PTSD does not end with the veteran. We recognize the critical role of the veteran’s family in supporting veterans’ well-being. Chronic conditions, such as comorbid pain and PTSD, can have substantial impacts on personal relationships. Studies have linked unhealthy family responses to poorer outcomes in the person with pain [27]. Maladaptive patterns of interaction may reinforce disability, fear of activity, and dependency in the patient, thereby inhibiting their recovery, rehabilitation, and treatment outcomes. Family members or other support partners may respond in a manner that is solicitous, thereby unintentionally reinforcing the sick role and disabled lifestyle of the person with pain [27]. Though partners often serve as a protective factor through social support and advocacy, problematic effects of caregiver burden are common [28,29]. Family and loved ones’ involvement in treatment can help support positive outcomes [30-32].

### Partner-Family–Assisted Interventions

Including partners-family in treatments has been conceptualized in four ways: (1) partner-assisted interventions, (2) disorder-specific interventions, (3) general therapy, and (4) education-facilitated engagement [33,34]. MR qualifies as a partner-family assisted intervention, as MR is not disorder-specific nor a focused couples therapy nor primarily educational. Instead, it involves providing guidance and encouragement to the partners or family members so they can support the veterans’ engagement in and experience of MR. The majority of family-partner–focused interventions focus on mental health and couples-family therapy [33,34]. MR is unique in its conjoint approach to supporting the veteran through the use of partnered CIH. A veteran-partnered sample participated in a multimodal intervention to address PTSD symptoms using CIH modalities for stress reduction and resource building. Findings indicate significant reduction in PTSD symptoms and benefits to their family members [35]. Though current research in partner-assisted CIH based programs is limited, these programs hold potential for supporting veterans with chronic conditions and their partners/families.

### Introduction of an Innovative Partner-Family–Assisted Complementary and Integrative Health Intervention

Mission Reconnect (MR) provides a novel CIH approach that supports veterans’ symptom management using evidence-based CIH modalities (ie, massage, meditation, positive psychology) presented in a self-paced partner-family–assisted program that can be accessed remotely. MR is distinctive in its:

1. Approach to treating chronic pain using a nonpharmacological CIH approach to managing chronic pain that has been shown successful in a community-based nonclinically defined cohort [36].
2. Use of CIH therapy skills education, which teaches massage techniques as a home-based, interpersonal skill between veterans and their selected partners.
3. Focus on the relationship dyad as the unit of intervention. The proposed *partner-family–assisted intervention* directly applies the biopsychosocial model, engaging veterans in their natural social contexts rather than relying solely on a clinical setting. Targeting the dyad leverages the natural resources of the relationship—trust, commitment, compassion, and mutual reinforcement of participation.

### Complementary and Integrative Health Impact on Pain and Posttraumatic Stress Disorder Outcomes

Massage is the most preferred CIH modality of all complementary health approaches, with broad appeal among veterans [37]. Research indicates 82% of veterans with chronic pain reported use of at least one CIH therapy and nearly all (99%) were willing to try such approaches [37]. These findings are consistent with military treatment facilities that report CIH is most often used use for pain and mental health conditions [38]. The preference for massage is intuitive given evidence suggesting the therapeutic benefits of massage including reduction of pain, stress, depressive symptoms, anxiety, and improvement of sleep across diverse populations [36,39-43]. A 2016 VA evidence-based synthesis report identified 21 high-quality systematic reviews on massage for pain. Findings described potential benefits of massage, but evidence strength is limited due to methods used in reviewed studies [44]. An independent meta-analysis of randomized controlled trials (RCTs) addressing pain concluded that massage effectively reduced pain compared to sham and active comparators, and improved mood and health-related quality of life compared to active comparators, and concluded that massage is a viable pain management option [45]. A limited number of massage studies have shown results on PTSD, but many studies have demonstrated results on related symptoms such as anxiety, stress, and depression [40,42,46-48].

Though limited research connects the impact of massage on PTSD outcomes, a secondary analysis of four trials with veterans with PTSD suggests that mindfulness-based stress reduction, another MR component, is a viable mechanism of treatment [49]. Mindfulness reduces pain for veterans with chronic pain [50] and improves anxiety, depression, and suicidal ideations [51]. Based on a VA review of the current evidence and best practices, VA/Department of Defense (DOD) PTSD clinical practice guidelines suggest that, although CIH is not indicated as primary treatment, it holds promise to improve wellness and promote recovery [52]. Even with these clinical guidelines, it is noted that study limitations leave current evidence inconclusive [52]. Another systematic review of CIH among veterans and military personnel indicated benefits from mind-body modalities; however, the quality of most RCTs was rated poorly [53]. The evidence base regarding the effectiveness of CIH interventions [44] for reducing pain and PTSD symptoms in veterans is inconclusive; this study will fill the gap in this area of research [54]. This project will contribute to the knowledge base of this field of research by using strong methodology related to sample size, contextual specificity of the target population, RCT design, and measurement of long-term outcomes.

## Methods

### Conceptual Framework

**Figure 1:**
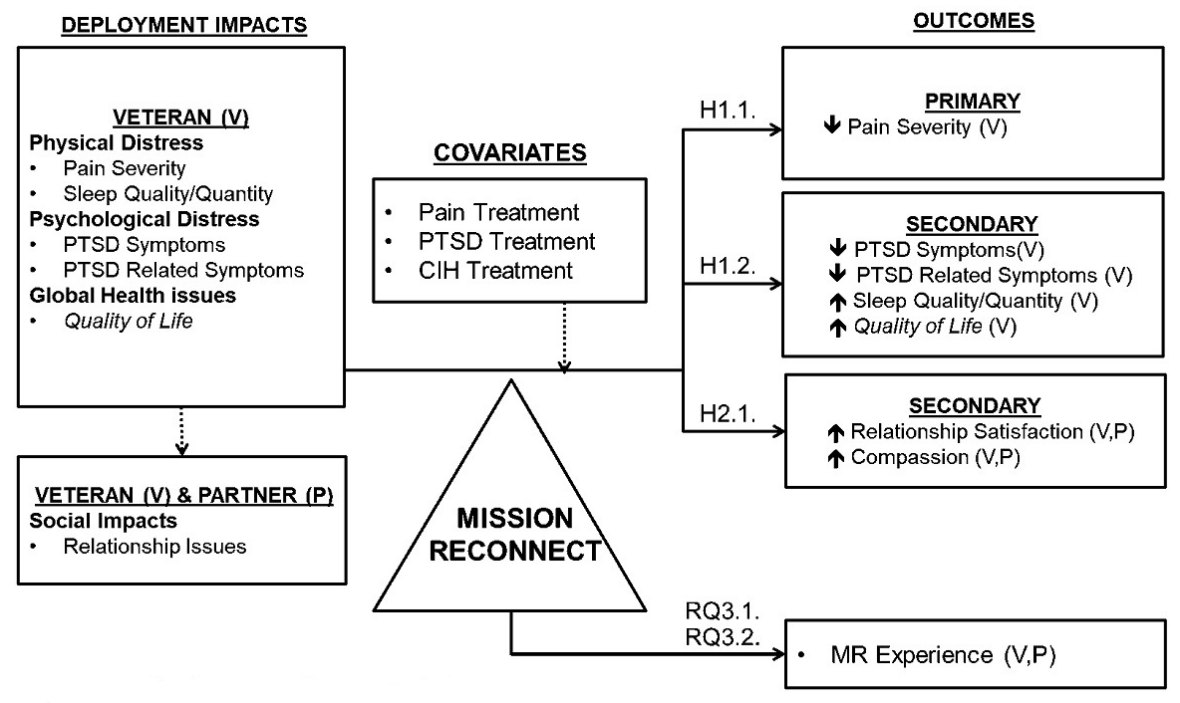
Conceptual model. CIH: complementary and integrative health; H: hypothesis; MR: Mission Reconnect; PTSD: posttraumatic stress disorder; RQ: research question.

In our conceptual model (Figure 1), deployment impacts on veterans and their families are summarized in the left box while target biopsychosocial outcomes in the boxes on the right are the expected effects of the proposed MR intervention, while controlling for previous and current treatment history (non-MR). MR has potential to provide a critically needed CIH option to manage deployment-related impacts, such as pain symptoms, sleep issues, and relationship issues.

### Hypothesized Mechanisms of Mission Reconnect

The biopsychosocial model was proposed to encourage exploration into categories of human distress that did not neatly fit the physiology-only, biomedical concept of disease and the processes by which disease developed [55]. The biopsychosocial model supports interdisciplinary programs that combine intervention components at the biological, psychological, and social levels [24,25]. MR leverages the mechanisms of action of multiple evidence-based CIH approaches, offering users multiple pathways to achieve clinical benefit. Users can benefit from the discrete therapies themselves but also the synergy of diverse therapies that mutually reinforce each other’s effects. The primary modalities taught in MR—cognitive and behavioral approaches such as activation of mindfulness, gratitude and compassion, and massage—have very rich evidence bases for reducing pain and anxiety. Finally, the interpersonal support conferred by collaborative participation in MR makes possible the buffering effects of social support while reinforcing practice activities [56]. Impacts of MR participation on pain have been demonstrated in both the Phase I and Phase II trials [57].

### Research Design and Methods

**Figure 2:**
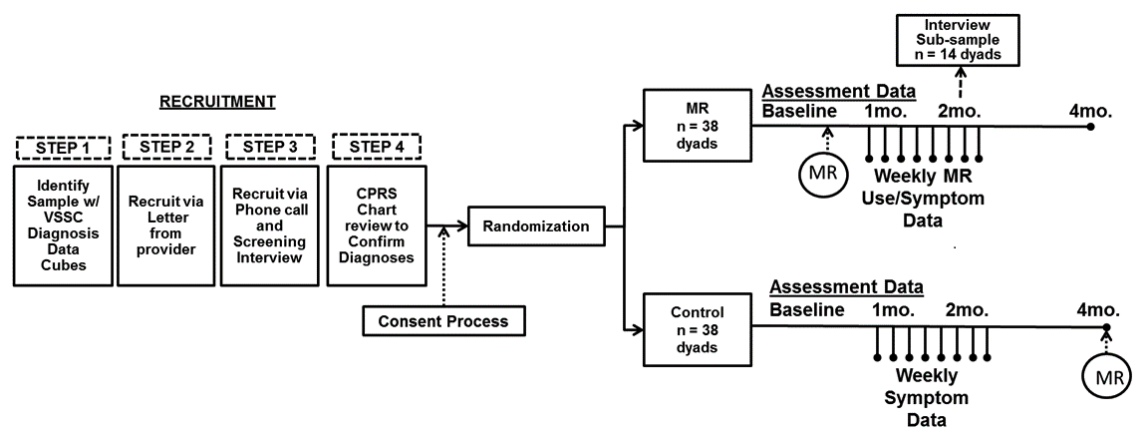
Study flow chart. and abbreviation CPRS: Computerized Patient Record System; MR: Mission Reconnect; VSSC: Veterans Health Administration Support Service Center Capital Assets.

This 4-year RCT with one intervention arm and one wait-list control arm will use mixed methods to evaluate the effectiveness and perceived value of the MR program in relation to physical and psychological symptoms, global health, and social outcomes. An underpinning of the MR program is that it is intended to be adjunctive (complementary) to conventional evidence-based modalities for treatment of pain and PTSD (ie, usual care). This is consistent with the previous MR trial conducted in a non-VA setting and different patient population whereby usual care served as the comparator group. Therefore, a waitlist control condition has been selected as the comparator for the proposed trial. This will allow assessment of the MR program above and beyond the effects of usual care being received. In addition, the relatively brief waitlist control period insures that all subjects will ultimately be offered the MR program. We believe this will be an incentive for both recruitment and retention of study subjects for this new recruitment setting. We will test MR using an RCT with concurrent mixed-methods data collection. We will recruit veteran and partner dyads. Study flow for each site is shown in Figure 2.

Aims 1 and 2 assessment data will be collected from 228 veteran/partner dyads (76 dyads per site, 38 dyads per arm) via a secure project website at baseline, 1, 2, and 4 months to assess primary and secondary outcomes. MR utilization and pain ratings will be assessed weekly for the first 8 weeks of the intervention for the treatment group. This data collection plan will allow assessment of acute changes, rate of change with repeated measures over 2 months and sustained changes.

In Aim 3, a subsample of 42 treatment group dyads (14 per site) will be purposively selected (high- vs low-volume MR use) to participate in telephone interviews to evaluate their experiences using the program. All consenting participants will be randomly assigned in a 1:1 ratio using a permuted variable block design to one of two arms (treatment vs control). Participants will receive study information, instructions on how to complete the online data collection, and contact information in case they experience an adverse event. Participants will provide preferred contact information for data collection reminders.

### Treatment Arm

MR is a user-driven, dyadic, self-care management program developed with the National Institute of Mental Health funding (R43/44) for use by veterans and their selected partners, individually or together, to reduce pain and distress and support physical, mental, and relationship health. MR was designed for veterans who face obstacles accessing formal mental health services. It can also be used to complement formal services. MR is a patient-centered intervention, allowing users to determine the pace at which to proceed in each program component.

### Mission Reconnect Content

The program provides video and audio instruction in a set of 11 evidence-based wellness activities in three thematic categories: Connecting with Yourself, Connecting with Quiet, and Connecting with Your Partner. All instruction is accessible via the Mission Reconnect website and mobile device apps. Video content totals 91 minutes and was produced by filming 2 days of workshops to teach the practices to veteran/partner dyads. The Program Overview video (54 minutes) introduces the MR instructional sequences accompanied by commentary by workshop participants. Detailed massage instruction is presented in the separate Massage Instruction video (34 minutes) and Massage Video Supplement (3 minutes) addressing use with home furniture. Users are encouraged to give and receive at least one massage per week. Audio content totals 67 minutes and was recorded in studio, with nine instructional audios ranging from 1-22 minutes. Users are encouraged to listen, learn the practices, and then use each technique with or without the guided instruction as they wish.

A *Massage Instruction Booklet* and illustrated *Massage Reminder Handout* are downloadable from the website. A *What if?* feature enables users to access advice on how to apply program techniques in challenging situations such as experiencing problems with sleep, focus, and concentration. Users can submit questions and suggestions for future content through the *What if?* interface. Optional Audios give users choice of audio gender voice. A Resources section provides links to hotlines, Vet centers, and VA facilities.

Users will be instructed to (1) try all the practices at least once during the first 2 weeks, and thereafter (2) do at least one exchange of massage per week with their partner, and (3) practice other methods of their choice at least 3-4 times per week. Dyads will be informed that greater use may result in greater benefit to reinforce practice.

### Usual Care Waitlist Control Arm

For ethical reasons, this study will use a waitlist control arm to ultimately offer all participants exposure to the MR intervention. Partner dyads in the control arm will participate in all assessments like those in the treatment group; however, they will be asked to agree to not access the public website during their participation. Study team will maintain participant engagement with this program through a variety of methods, including email blasts, reminder prompts, and marketing blasts. Wait-list control participants will be instructed to seek advice about treatment from their providers. Other than this initial advice, there will be no attempt by study personnel to influence condition management unless an issue arises (eg, suicidal ideation). The control condition will account for potential temporal effects that occur from passage of time (brief) and expectation effects associated with anticipation of MR participation. While dyad randomization suggests primary physicians will have intervention and control patients in their practices, numerous effectiveness trials have shown there is little spillover of the intervention to usual care patients. The control group will receive access to MR after they complete data collection.

### Sampling

We will use a four-step process to purposively recruit study participants (Figure 2). First, using an Institutional Review Board–approved procedure with a of waiver of consent process; a secondary administrative data query of the *International Classification of Diseases, Ninth and Tenth Revisions* (ICD 9-10) conditions for chronic pain and PTSD will identify a sample pool of veterans in the previous fiscal year. With our access levels and expertise, this process should take 2 weeks. Second, we will confirm approval to recruit from providers using signed letters from veterans’ providers. Providers are being notified about study details and will provide recruitment letters to patients for them to follow up with a study team member about participating in the study.

Screening interviews will assess the occurrence of chronic pain (ie, pain for 6 months or more during the prior year) and symptoms of PTSD (ie, diagnosed, treated, or experienced symptoms of PTSD in the past 6 months); assess if they have visual, hearing, or other cognitive impairments; assess for previous diagnosis of moderate or severe traumatic brain injury (TBI); recent history of psychosis; and determine availability of a partner and the dyad’s interest in participation. Additional details on pain treatment history, severity and treatments of PTSD symptoms, potential TBI exposure, and recent use of complementary and alternative therapies for pain will be captured from the baseline data collection forms. In tandem, the fourth step (with waiver of consent) will allow chart review if needed for interested potential participants to (1) confirm comorbid conditions (ie, chronic pain and PTSD), (2) determine substance use disorder treatment status, and (3) determine TBI diagnosis and severity to exclude individuals with moderate or severe TBI.

Eligible veterans will be invited to participate after screening, be consented, and receive information to access the data collection site and determine their group assignment. Veterans and their partners will receive separate stipends via mail for their time.

The sample will consist of 152 participants (76 dyads) over approximately 24 months at each site. Women and minorities recruitment numbers will mirror site distribution based on sex and race, as this study is not powered to examine differences by race and sex. We anticipate considerable racial variability due to having three geographically diverse sites with markedly different census profiles. We anticipate a considerable representation of female participants with our invested partnership with a women’s physician. This will make a considerable contribution to the knowledge of female veterans which is often lacking in VA studies. Recognizing the potential limitation of this strategy given the inclusion/exclusion criteria we, will also gain Institutional Review Board approval to collaborate with the local Rehabilitation Outcomes Research Section Veteran Engagement Council and co-investigators to recruit veteran participants using other recruitment means such as referrals and advertisements (eg, poster, brochures).

Inclusion criteria for participants will comprise the following: age 18 years or older, English-speaking veterans, and chronic musculoskeletal pain [58] as initially identified through secondary administrative data query of the ICD-9-10 conditions for chronic pain. In the initial query, musculoskeletal pain is present if the veteran meets either of two validated criteria: (1) ≥2 occurrences of any of targeted musculoskeletal *International Classification of Diseases and Related Health Problems, Ninth Revision, Clinical Modification* (ICD-9-CM) codes “likely to represent chronic pain” identified by Tian et al [59] recorded at visits separated by at least 30 days within past 6 months; or (2) high impact chronic pain equal to ≥2 occurrences of targeted musculoskeletal ICD-9-CM codes (adapted from Goulet et al [4]) separated by at least 30 days within the 6 months prior to study recruitment *and* ≥2 pain scores greater ≥4 separated by at least 30 days within past 6 months [59]. For pain scores, we will use the 0-10 numeric pain rating scale that is routinely collected at the VA. We will count two ICD-9-CM codes or pain scores recorded on the same day as one code/score.

Previous history of PTSD will be present if the veteran has a flag in their medical record indicating confirmed condition by the VA Compensation and Benefits program, has at least two outpatient visits in the year with the primary diagnosis being listed as PTSD (ICD-9-CM code 309.81), and/or had PTSD listed on the problem list. Confirmation of chronic pain for more than 6 months in the past year and diagnosis, treatment, or symptoms of PTSD in the past 6 months will be confirmed by the telephone screening interview and use of the Computerized Patient Record System if needed for further confirmation. To participate in the study, the veteran must also have ability to access and use an electronic platform (eg, mobile device, Internet, DVD) for MR delivery, with a willing partner to participate in the study and MR program.

Exclusion criteria will include the following: moderate to severe TBI; diagnosis or documented treatment for psychosis in previous 6 months; currently in high-intensity substance use disorder treatment; non-English speaking; visual, hearing, cognitive impairment that prevents participation or ability to consent; self-report history of partner/family member physical abuse in the past year; and/or lack of Internet access. As stated above, potential participants who screen for aggression or violence will also be excluded from study.

### Informed Consent

A waiver of informed consent process for recruitment purposes (medical record review) and a waiver of consent for a Verbal Consent Document for participant phone screening will occur. The study team will also be using a Written Consent Document or verbal consent via telephone.

Participants will complete the informed consent and Health Insurance Portability and Accountability Act (HIPAA) authorization for the study either in-person or over the phone. Research team members will review the consent and HIPAA content with participants to ensure review and comprehension. Since both partners will be full and equal participants in the study, both will be individually screened and consented by individual interview. We will employ three self-report items to address physical threat to and by partner, and fear reprisal. We will use a standard protocol advised and used in VA Family Services in its couples/family therapy: (1) initial brief – individual consent/self-report, (2) identify urgent need, (3) provide follow-up call and referral for community resources, and (4) exclude from study.

Participants will be provided an option to receive a printed copy of their informed consent and HIPAA authorization for review to ensure their understanding. Communication, such as informed consent explanations, will be supplemented using the phone to provide respondents opportunities for questions and clarification as needed. This is an effective means of communication for studies evaluating electronic health services such as MR. These remote methods of communication and consent have been effectively used in other studies conducted by the PI. These forms of communication (1) reduce participant burden, (2) conserve resources, and (3) leverage electronic communication devices, which support and promote virtual care.

### Randomization

Participants (partner dyads) will be randomized to treatment (MR) or control using a per site variable block randomization method (blocks of 6 and 8) to facilitate a balance in treatment and control group sizes per site and over time. Variable blocks of 6 and 8 per site will be randomly used so that no one will know for sure the next random assignment. Dyad is the unit of randomization; this ensures that members of the same couple receive the same intervention. The randomization will be stratified by whether or not the veteran is currently receiving, or has received in the past 2 weeks, a Level 1 evidence-based treatment for pain and/or PTSD, per current VA-DoD treatment guidelines.

This will facilitate subgroup analyses of the effect of the MR intervention in the presence versus absence of concomitant first-line evidence-based treatment for pain and PTSD. A computer-generated random allocation sequence will be generated by the study biostatistician, separately for each site/era of service combination to ensure balance across sites (ie, control for site effects by design). The sequence will be concealed to participants until immediately after their consent and baseline data collection, when group assignment information will be revealed on the last page of baseline survey. Participant dyads randomized to the treatment group will receive a link and sponsor code to access the MR site and will be instructed to create personal user accounts. Control group members will receive access to MR after completing data collection.

### Sample Size Power Analysis

Estimates of statistical power are based on Aim 1 and initially the primary outcome of pain. Whereas dyad is the unit of randomization, we assess power based on separate analyses for veterans and partners (one approach used). There are four major intervals of assessment (baseline, 1, 2, and 4 months). For the 228 dyads, we conservatively assume up to 20% missing data over follow-up despite the previous MR trial that had less than 5% attrition. Assuming within-subject (outcome measurement) correlation of .5, the proposed sample size will provide 80% power (2-sided type I error rate of 0.05) to detect a “small-to-medium” effect size of 0.38 (Cohen *d*). For analyses stratified by site (in addition to evaluation by random effect assessment), the study will provide 80% power to detect a “medium-to-large” effect size of 0.66. By way of comparison, the pilot data presented above describe large effect sizes (*d*=0.81 for pain symptom reduction). While this effect size was based on a relatively small sample of comparable patients (N<15) and within-subject assessment (paired *t* test), it provides good assurance that the proposed sample size is sufficient to detect realistic, medium size effects should they exist with the MR program. For secondary outcomes, we will use a type I error rate of 0.01 for multiple comparisons. For the sample and our described assumptions, this will provide 80% power to detect a medium effect size of 0.45 [60].

### Sample Size

Qualitative Aim 3 sample size relies on the quality and richness of information obtained [61]. Conceptual saturation is the goal of qualitative research and depends on data to support interpretations. Saturation has been noted to occur within the first 12 interviews [61,62]. Our team has extensive experience in evaluating electronic technologies and has found that recruiting high- and low-volume users provides the richest dataset. To ensure representative data, we will conduct telephone interviews with a purposive (high- vs low-volume users) subsample of up to 42 dyads to achieve representation at each site (14 dyads/site).

### Data Collection Procedures

To test Aims 1 and 2, survey data will be collected using Qualtrics, a resource that has demonstrated capacity for remotely and securely collecting participant data in other studies conducted within the VA system. Qualtrics accounts are password-protected, and all data are replicated in real-time. The participants will be assigned a unique personal identification number (PIN) and will receive email messages with a link to prompt participant dyads to access the site and complete data collection. Upon access to Qualtrics, participants will be required to enter their PIN as the first entry into the system. Similarly, access to the online MR content will require entry of the same PIN. The PIN selected by each participant will be maintained in a cross-walk file with their randomly assigned study ID number. The Qualtrics measures will be compiled into a single survey format and collected at baseline, 1, 2, and 4 months. The rationale for this schedule is to formally assess initial, short-term, and sustained effects that may occur with the MR intervention. MR use and satisfaction, pain, tension, and stress items will be conducted weekly for the first 8 weeks of data collection. This more frequent schedule of data collection will permit short-term dose-response analyses (ie, dose of MR utilization) in relation to major symptoms of pain and PTSD. Measures and psychometric properties are illustrated in Table 1 [63-76].

To address Aim 3, telephone interviews (approximately 30 minutes) will be conducted with a subsample of 42 dyads, one month after the onset of the intervention (MR group). Only treatment group members will be recruited for this data collection. Interviews will be conducted with veterans and their partners separately. We will explain the interview purpose, ask permission to audiorecord, and use the interview script to ensure that all topics are covered. Interviewers will solicit respondents’ attitudes, opinions, and reports about their preferences and the pros and cons of MR and participating in the practice groups, including their perceptions of usefulness. We will use standard communication techniques to stimulate discussion, with prompts (eg, “tell me more”), summarizing statements, and silence. We have used these methods in several previous studies to gather data effectively.

**Table 1:** Veteran and partner measures and psychometric properties.

Variable, construct		Measure	Description	Minutes	Time point
**Veteran and partner independent variables**					
	Demographics	Participant survey	15 items assessing age, gender, ethnicity/race, education, income levels, religion, marital status, relationship length, military service/status/grade, deployments, computer/internet use	5	Baseline
**Veteran independent variable: covariate**					
	Other treatments	Concurrent treatments	2 items asking veterans to report concurrent pain and PTSD^a^ treatment(s) and CIH^b^ modalities to account for dual intervention effects	1	Baseline, Months 1, 2, 4
**Veterans and partner independent variable: covariate**					
	MR^c^ program utilization (treatment and control group)	Utilization survey	11 items assessing frequency of use of the MR mind/body and massage practices	3	Weeks 1-8; Months 1, 2, 4
**Veteran dependent variables**					
	Quality of life	Self-Assessment of Change [63,64]	16-item word-pairing scale assessing a variety of shifts in well-being across a broad range of therapeutic modalities and conditions	5	Months 1, 2, 4
	Quality of life	Quality of Life Short Form-12 [65]	12 items assessing quality of life using physical status and mental health distress	3	Baseline, Months 1, 2, 4
	Pain	Defense and Veterans Pain Rating Scale [66]	Pain Numeric Rating Scale [67], 11-point scale measuring “usual” pain intensity over last week and 4 pain functionality (past month) items	3	
		Pain Outcomes Questionnaire - Short Form VA^d^ [68]	19 items assessing pain-related domains, including pain intensity, interference with activities and mobility, negative affect, vitality, pain-related fear; and improbable pain-related symptoms	5	
	TBI^e^ exposure	OHIO^f^ TBI Exposure Screen [69]	8 items designed to elicit self- or proxy-reports of TBI occurring over a person’s lifetime; can provide measures of extent of exposure to TBI including current injury	5	Baseline
	Pain	Single-item scale	One item assessing pain using a 0-5–point Likert-type scale	1	Baseline, Weeks 1-8; Months 1, 2, 4
	Stress and tension	Single-item scale	Two items assessing stress and tension using a 0-5–point Likert-type scale	1	
	PTSD & related psychological symptoms	PTSD Checklist [70]	20-item measure of the *Diagnostic and Statistical Manual of Mental Disorder, Fifth Ed* (DSM-5) PTSD symptoms with scales related to stress, anxiety, & emotional numbing	4	Baseline, Months 1, 2, 4
		Depression: Beck Depression Inventory [71]	21 items, a widely used instrument for measuring depression; respondents asked to rate their symptoms and attitudes using a 4-point scale	5	
		Stress: Perceived Stress Scale [72]	10 Likert-scaled items, validated and widely used, to determine perceived stress levels	3	
		Sleep Quality: Pittsburg Sleep Quality Index [73]	19 self-rated questions from which 7 component scores are calculated and summed into a global score to assess sleep quality in the past month	4	
**Veterans and partner dependent variables**					
	Relationship satisfaction	Revised Dyadic Adjustment Scale [74]-Adapted	14-item Likert-scaled instrument is reliable and valid and contains subscales for dyadic consensus, dyadic satisfaction, and dyadic cohesion.	3	Baseline, Months 1, 2, 4
	Compassion	Compassion for self and others scales [75,76]	26-item Self-Compassion Scale and 21-item Compassion for Others measures, using 5-point Likert scale	5	
	Program satisfaction (treatment only)	MR program satisfaction items	Eleven 10-point Likert-type items assessing satisfaction using MR components, whether they would recommend MR, and massage satisfaction	1	Weeks 1-8; Months 1, 2, 4

^a^PTSD: posttraumatic stress disorder.

^b^CIH: complementary and integrative health.

^c^MR: Mission Reconnect

^d^VA: Veterans Affairs

^e^TBI: traumatic brain injury.

^f^OHIO: Ohio State University TBI Identification Method.

### Data Analysis

The intention-to-treat principle will be used for all analyses regardless of the extent of protocol compliance or dropout [77]. Whereas randomization is anticipated to balance both study arms on presenting demographic and clinical characteristics, including within sites and by concomitant receipt of first-line pain or PTSD treatment, continuous variables will be compared by student *t* tests or Wilcoxon tests (depending on distributional properties); categorical variables will be compared by chi-square analyses.

All outcome measures (primary and secondary) under Aim 1 will be collected at baseline, 1, 2, and 4 months and are continuous variables. Therefore, general linear mixed models will be used with main effects terms for GROUP (MR vs waitlist) and TIME (4 time points) and a GROUP x TIME interaction term (for rate of change). The models will also include site as a random effect. To assess whether the rate of change is curvilinear (ie, the rate of change differs between time points), a quadratic parameter will be tested. Different correlation structures and functional forms of the effects of the MR program will be assessed using the information criteria and a final parsimonious model will be determined for final statistical inference. Initial effects of the MR program (baseline to 1 month) will be evaluated by analysis of covariance. The primary outcome measure for pain will be the total score on the 19-item Pain Outcomes Questionnaire. The primary outcome measure for PTSD will be the total score on the 20-item PTSD Checklist for DSM-5. Subgroup analyses will be explored using similar methods and examination of severity of baseline pain and PTSD scores. These analyses will provide insight into subgroups for whom the MR program may be particularly beneficial. In a similar realm, analyses will be stratified by baseline median level of relationship satisfaction, as derived from total score on the 14-item Revised Dyadic Adjustment Scale. This will permit assessment of the effect of the MR intervention on potential improvement in relationship satisfaction among a cohort of dyads with presumably troubled relationships at entry. Moreover, using the weekly reports (8 weeks) of MR use and satisfaction with the MR program, the MR sample will be split above versus below the median for these two measures and compared separately against the waitlist control condition at 2 months. This approach approximates a “per protocol” analysis in terms of recommended use of the MR program. In addition, especially for Aim 2 outcomes, we seek to examine whether dyads appear to show mutual benefits from the MR program. Therefore, multilevel models will be fit using an over-time dyadic model [78] in which individuals are nested within dyads and time is crossed with dyads (ie, both veteran and partner are assessed at the 4 time points). This analysis accounts for the non-independence due to the correlation between dyad members’ general levels on outcomes averaged over time, as well as the time-specific correlation between their outcomes (eg, similarity caused by time-specific events).

Interview transcript data will be managed using descriptive content analytical methods to identify domains and taxonomies related to participants’ experiences with MR use [61]. Categories will be compared and contrasted, and relationships among them will be identified. As coding schemas are developed to create domains and taxonomies, data samples will be extracted and coded by research team members and evaluated for interrater reliability and credibility. Descriptive and comparative matrices, which identify the patterns of regularities (shared) and inconsistencies (unique or varied) will then be constructed for the veteran and partner participants. Comparative matrices enable identification of the most relevant, shared, and perhaps representative components, thereby enhancing the potential representation of the findings. Finally, a complex cross-case data display or matrix will be developed to summarize the significant taxonomic outcome structures identified within and between veterans and their partners. This process of descriptive and comparative matrix analysis will allow discernment of the most salient and representative components identified by veterans and their partners.

### Missing Data

Missing data will be tabulated by treatment arms and by assessment waves; comparison tests between arms will be conducted to assess potential attrition bias and to examine the missing data mechanism (eg, missing at random). If the missing rate is less than 10%, analyses with list wise deletion (ie, missing values dropped from the analysis) will be performed due to minimal concern over bias. Participants who are lost to follow-up and missing on postrandomization outcome assessments will be included in additional comparison analyses that use multiple imputations of missing data to minimize bias due to differences between those with complete and incomplete data.

## Results

Funding for the study began in November 2018, and we are currently in the process of recruitment screening and data randomization for the study. Primary data collection will begin on May 2019 and continue through May 2021. Projected participants per site will be 76 partner dyads, for a total of 456 study participants. Anticipated study results will be published in November 2022. Table 2 shows the projected study schedule.

**Table 2:** Gantt chart of study benchmarks.

Project activity	Year 1 (2019-2020)				Year 2 (2020-2021)				Year 3 (2021-2022)				Year 4 (2022-2023)			
	Q1 Nov ’18	Q2 Feb ’19	Q3 May	Q4 Aug	Q1 Nov	Q2 Feb ’20	Q3 May	Q4 Aug	Q1 Nov	Q2 Feb ’21	Q3 May	Q4 Aug	Q1 Nov	Q2 Feb ’22	Q3 May	Q4 Aug
Start-up	X^a^															
Recruitment screening and randomization, Qualtrics development and validation		X														
Recruitment: 3-4 dyads/month per site			X	X	X	X	X	X	X	X						
Primary data collection			X	X	X	X	X	X	X	X	X					
Conduct interviews				X	X	X	X									
Interview transcription				X	X	X	X	X								
Interview data analysis					X	X	X	X	X							
Prepare and stage primary data										X	X	X				
Primary data analysis													X	X		
Data interpretation and triangulation													X	X	X	
Finalize data reports/manuscripts															X	X
Develop materials for dissemination															X	X
Prepare/submit subsequent proposal															X	X
Disseminate materials to audiences															X	X

^a^X: denotes the activity occurred during this time frame.

## Discussion

### Principal Considerations

The goal of this study protocol is to use evidence-based CIH methods to decrease chronic pain and PTSD symptoms in the veteran-partner dyad. The research goal is to evaluate MR as an approach to manage chronic pain and PTSD symptoms for potential subsequent implementation. This study will possibly provide a model for establishing remote access and sustainable implementation of CIH within VA. If shown to be effective, there is reason to believe it is scalable, feasible, and sustainable and can ramp up nationally. This will also relieve a bottleneck of services by moving from a provider-based delivery to partner delivery.

### Strengths and Limitations

This protocol contributes to the science in three distinct ways: (1) a conjoint approach to supporting the veteran through the use of partnered CIH, (2) use of a self-directed evidence-based CIH mobile app for a clinically defined population of veterans and partner, and (3) MR’s distinctive use of nonpharmacological interventions to manage chronic pain.

This protocol is an RCT approach powered for generalizability. Mixed methods will illuminate the how and why of the veterans’ and their partners’ experience of mobile apps for CIH. The strength of the intervention is that it is remotely delivered thus overcoming geographical restrictions. If shown to be effective, it can be scalable and will relieve potential service bottlenecks by establishing partner-based delivery rather than provider-based delivery.

A limitation in the analytic methods are threats to protocol by using remote access in an electronic model, which may have an impact on recruitment, attrition, and potential issues in the delivery process. However, safeguards are in place, which include staff to help troubleshoot. Using remote data collection can be challenging. By taking this protocol away from paper-based responses and into a model that leverages mobile and remote data collection, lessons may be learned on how best to exercise remote and electronic data collection methods.

The limitations in waitlist controls have been criticized due to ethical concerns. A waitlist control is not ethical when there are other treatment and interventions available. The waitlist can speed recruitment and parcel out expectations, and people may get better anyway. It is a control condition which is better than no control, and it is appropriate at an early stage of intervention development. This protocol allows waitlist controls to gain exposure and provide the study with a comparator group of the proposed trial. The attractive part of this waitlist is knowing they will get the intervention.

The study scope is focused on providing a remote intervention with veterans who have PTSD and pain symptoms. Though veterans who are symptomatic and not engaged in the health care system may benefit from this intervention, they are not the target of this protocol. It is also possible veterans who are symptomatic but not documented in the VA system may also be missed due to using secondary data source to identify the sample pool. The method of recruitment using secondary sources was based on optimal feasibility for large-scale RCT recruitment. Future research should focus on use of mobile app technology to improve access to CIH in a variety of populations. Additionally, future research should look to the most appropriate electronically captured patient-reported outcome tools. This research did not take the opportunity to measure the partners’ outcomes due to the funding source mechanism. Therefore, future research should look at partner experience. If highly variable responses/outcomes with MR are found, then data that include participant characteristics will guide intervention modifications for this patient group.

### Conclusions

This intervention is extremely important and innovative. Large government organizations currently have limited capacity for electronic patient-reported outcomes. Patient-reported outcomes and MR can test different measures electronically by optimizing use of technology and remote delivery of intervention and data collection methods. From the user perspective, MR allows the user to leverage a “when they want, where they want” approach. Optimizing use of technology and remote delivery of intervention and data collection methods will contribute to the field of science.

## Appendix

Multimedia Appendix 1 Funding reviewer comments.

## Abbreviations

CIH: complementary and integrative health
DOD: Department of Defense
DSM-5: Diagnostic and Statistical Manual of Mental Disorders, Fifth Edition
ICD-9-10: International Classification of Diseases and Related Health Problems, Ninth and Tenth Revisions
ICD-9-CM: International Classification of Diseases and Related Health Problems, Ninth Revision, Clinical Modification
MR: Mission Reconnect
PTSD: posttraumatic stress disorder
RCT: randomized controlled trial
VA: Veterans Affairs
VHA: Veterans Health Administration

## References

[1] Lew HL, Otis JD, Tun C, Kerns RD, Clark ME, Cifu DX (2009). Prevalence of chronic pain, posttraumatic stress disorder, and persistent postconcussive symptoms in OIF/OEF veterans: polytrauma clinical triad. J Rehabil Res Dev.

[2] Kip KE, Rosenzweig L, Hernandez DF, Shuman A, Diamond DM, Girling SA, Sullivan KL, Wittenberg T, Witt AM, Lengacher CA, Anderson B, McMillan SC (2014). Accelerated Resolution Therapy for treatment of pain secondary to symptoms of combat-related posttraumatic stress disorder. Eur J Psychotraumatol.

[3] Goulet JL, Erdos J, Kancir S, Levin FL, Wright SM, Daniels SM, Nilan L, Justice AC (2007). Measuring performance directly using the veterans health administration electronic medical record: a comparison with external peer review. Med Care.

[4] Goulet JL, Kerns RD, Bair M, Becker WC, Brennan P, Burgess DJ, Carroll CM, Dobscha S, Driscoll MA, Fenton BT, Fraenkel L, Haskell SG, Heapy AA, Higgins DM, Hoff RA, Hwang U, Justice AC, Piette JD, Sinnott P, Wandner L, Womack JA, Brandt CA (2016). The musculoskeletal diagnosis cohort: examining pain and pain care among veterans. Pain.

[5] Seal KH, Cohen G, Waldrop A, Cohen BE, Maguen S, Ren L (2011). Substance use disorders in Iraq and Afghanistan veterans in VA healthcare, 2001-2010: Implications for screening, diagnosis and treatment. Drug Alcohol Depend.

[6] Seal KH, Shi Y, Cohen G, Cohen BE, Maguen S, Krebs EE, Neylan TC (2012). Association of mental health disorders with prescription opioids and high-risk opioid use in US veterans of Iraq and Afghanistan. JAMA.

[7] Ennis N, Sijercic I, Monson CM (2018). Internet-Delivered Early Interventions for Individuals Exposed to Traumatic Events: Systematic Review. J Med Internet Res.

[8] Kessler RC, Chiu WT, Demler O, Merikangas KR, Walters EE (2005). Prevalence, severity, and comorbidity of 12-month DSM-IV disorders in the National Comorbidity Survey Replication. Arch Gen Psychiatry.

[9] Hickling EJ, Blanchard EB (1992). Post-traumatic stress disorder and motor vehicle accidents. Journal of Anxiety Disorders.

[10] Sharp TJ, Harvey AG (2001). Chronic pain and posttraumatic stress disorder: mutual maintenance?. Clin Psychol Rev.

[11] Department of Veterans Affairs (2007). PTSD: National Center for PTSD.

[12] Tsang A, Von Korff M, Lee S, Alonso J, Karam E, Angermeyer MC, Borges GLG, Bromet EJ, Demytteneare K, de Girolamo G, de Graaf R, Gureje O, Lepine J-P, Haro JM, Levinson D, Oakley Browne MA, Posada-Villa J, Seedat S, Watanabe M (2008). Common chronic pain conditions in developed and developing countries: gender and age differences and comorbidity with depression-anxiety disorders. J Pain.

[13] Clark ME, Walker RL, Gironda RJ, Scholten JD (2009). Comparison of pain and emotional symptoms in soldiers with polytrauma: unique aspects of blast exposure. Pain Med.

[14] Smeeding SJW, Bradshaw DH, Kumpfer KL, Trevithick S, Stoddard GJ (2011). Outcome evaluation of the Veterans Affairs Salt Lake City Integrative Health Clinic for Chronic Nonmalignant Pain. Clin J Pain.

[15] Shi L, Liu J, Zhao Y (2012). Comparative effectiveness in pain-related outcomes and health care utilizations between veterans with major depressive disorder treated with duloxetine and other antidepressants: a retrospective propensity score-matched comparison. Pain Pract.

[16] Lang KP, Veazey-Morris K, Andrasik F (2014). Exploring the role of insomnia in the relation between PTSD and pain in veterans with polytrauma injuries. J Head Trauma Rehabil.

[17] Kupersmith J, Lew HL, Ommaya AK, Jaffee M, Koroshetz WJ (2009). Traumatic brain injury research opportunities: results of Department of Veterans Affairs Consensus Conference. J Rehabil Res Dev.

[18] Bosco MA, Murphy JL, Clark ME (2013). Chronic pain and traumatic brain injury in OEF/OIF service members and Veterans. Headache.

[19] Schnurr PP, Lunney CA (2012). Work-related outcomes among female veterans and service members after treatment of posttraumatic stress disorder. Psychiatr Serv.

[20] Westanmo A, Marshall P, Jones E, Burns K, Krebs EE (2015). Opioid Dose Reduction in a VA Health Care System--Implementation of a Primary Care Population-Level Initiative. Pain Med.

[21] Walker RL, Clark ME, Sanders SH (2010). The “Postdeployment multi-symptom disorder”: An emerging syndrome in need of a new treatment paradigm. Psychological Services.

[22] Ohye BY, Brendel RW, Fredman SJ, Bui E, Rauch PK, Allard MD, Pentel KZ, Simon NM (2015). Three-generation model: A family systems framework for the assessment and treatment of veterans with posttraumatic stress disorder and related conditions. Professional Psychology: Research and Practice.

[23] Capehart B, Bass D (2012). Review: managing posttraumatic stress disorder in combat veterans with comorbid traumatic brain injury. J Rehabil Res Dev.

[24] Engel GL (1980). The clinical application of the biopsychosocial model. Am J Psychiatry.

[25] Gatchel RJ, Peng YB, Peters ML, Fuchs PN, Turk DC (2007). The biopsychosocial approach to chronic pain: scientific advances and future directions. Psychol Bull.

[26] Geisser ME, Roth RS, Bachman JE, Eckert TA (1996). The relationship between symptoms of post-traumatic stress disorder and pain, affective disturbance and disability among patients with accident and non-accident related pain. Pain.

[27] Raichle KA, Romano JM, Jensen MP (2011). Partner responses to patient pain and well behaviors and their relationship to patient pain behavior, functioning, and depression. Pain.

[28] Nelson BS, Wright DW (1996). Understanding and treating post‐traumatic stress disorder symptoms in female partners of veterans with PTSD. Journal of Marital and Family Therapy.

[29] Verbosky SJ, Ryan DA (1988). Female partners of Vietnam veterans: stress by proximity. Issues Ment Health Nurs.

[30] Keefe F J, Caldwell D S, Baucom D, Salley A, Robinson E, Timmons K, Beaupre P, Weisberg J, Helms M (1996). Spouse-assisted coping skills training in the management of osteoarthritic knee pain. Arthritis Care Res.

[31] Abbasi M, Dehghani M, Keefe FJ, Jafari H, Behtash H, Shams J (2012). Spouse-assisted training in pain coping skills and the outcome of multidisciplinary pain management for chronic low back pain treatment: a 1-year randomized controlled trial. Eur J Pain.

[32] Bolton RE, Fix GM, VanDeusen Lukas C, Elwy AR, Bokhour BG (2018). Biopsychosocial benefits of movement-based complementary and integrative health therapies for patients with chronic conditions. Chronic Illn.

[33] Baucom DH, Shoham V, Mueser KT, Daiuto AD, Stickle TR (1998). Empirically supported couple and family interventions for marital distress and adult mental health problems. J Consult Clin Psychol.

[34] Monson CM, Macdonald A, Brown-Bowers A (2012). Couple/family therapy for posttraumatic stress disorder: review to facilitate interpretation of VA/DOD Clinical Practice Guideline. J Rehabil Res Dev.

[35] Church D, Brooks AJ (2014). CAM and energy psychology techniques remediate PTSD symptoms in veterans and spouses. Explore (NY).

[36] Kahn JR, Collinge W, Soltysik R (2016). Post-9/11 Veterans and Their Partners Improve Mental Health Outcomes with a Self-directed Mobile and Web-based Wellness Training Program: A Randomized Controlled Trial. J Med Internet Res.

[37] Denneson LM, Corson K, Dobscha SK (2011). Complementary and alternative medicine use among veterans with chronic noncancer pain. J Rehabil Res Dev.

[38] Herman PM, Sorbero ME, Sims-Columbia AC (2017). Complementary and Alternative Medicine Services in the Military Health System. J Altern Complement Med.

[39] Peterson K, Anderson J, Ferguson L, Mackey K Evidence Brief: The Comparative Effectiveness of Selected Complementary and Integrative Health (CIH) Interventions for Preventing or Reducing Opioid Use in Adults with Chronic Neck, Low Back, and Large Joint Pain.

[40] Haun JN, Graham-Pole J, Shortley B (2009). Children with cancer and blood diseases experience positive physical and psychological effects from massage therapy. Int J Ther Massage Bodywork.

[41] Cherkin DC, Eisenberg D, Sherman KJ, Barlow W, Kaptchuk TJ, Street J, Deyo RA (2001). Randomized trial comparing traditional Chinese medical acupuncture, therapeutic massage, and self-care education for chronic low back pain. Arch Intern Med.

[42] Moyer CA, Rounds J, Hannum JW (2004). A meta-analysis of massage therapy research. Psychol Bull.

[43] Tsao JCI (2007). Effectiveness of massage therapy for chronic, non-malignant pain: a review. Evid Based Complement Alternat Med.

[44] Evidence-based Synthesis Program (ESP) Center (2016). Evidence Synthesis Program.

[45] Boyd C, Crawford C, Paat CF, Price A, Xenakis L, Zhang W, Evidence for Massage Therapy (EMT) Working Group (2016). The Impact of Massage Therapy on Function in Pain Populations-A Systematic Review and Meta-Analysis of Randomized Controlled Trials: Part II, Cancer Pain Populations. Pain Med.

[46] Sharpe PA, Williams HG, Granner ML, Hussey JR (2007). A randomised study of the effects of massage therapy compared to guided relaxation on well-being and stress perception among older adults. Complement Ther Med.

[47] Moraska A, Pollini RA, Boulanger K, Brooks MZ, Teitlebaum L (2010). Physiological adjustments to stress measures following massage therapy: a review of the literature. Evid Based Complement Alternat Med.

[48] Haun J, Patel N, Schwartz G, Ritenbaugh C (2015). Evaluating the use of gas discharge visualization to measure massage therapy outcomes. J Complement Integr Med.

[49] Stephenson KR, Simpson TL, Martinez ME, Kearney DJ (2017). Changes in Mindfulness and Posttraumatic Stress Disorder Symptoms Among Veterans Enrolled in Mindfulness-Based Stress Reduction. J Clin Psychol.

[50] Garland EL, Manusov EG, Froeliger B, Kelly A, Williams JM, Howard MO (2014). Mindfulness-oriented recovery enhancement for chronic pain and prescription opioid misuse: results from an early-stage randomized controlled trial. J Consult Clin Psychol.

[51] Serpa JG, Taylor SL, Tillisch K (2014). Mindfulness-based stress reduction (MBSR) reduces anxiety, depression, and suicidal ideation in veterans. Med Care.

[52] Department of Veterans Afairs; Department of Defense (2017). VA/DoD Clinical Practice Guidelines.

[53] Elwy AR, Johnston JM, Bormann JE, Hull A, Taylor SL (2014). A systematic scoping review of complementary and alternative medicine mind and body practices to improve the health of veterans and military personnel. Med Care.

[54] Kligler B (2017). Research Advisory Committee on Gulf War Veterans' Illnesses.

[55] Engel GL (2012). The need for a new medical model: a challenge for biomedicine. Psychodyn Psychiatry.

[56] Cohen S, Wills T A (1985). Stress, social support, and the buffering hypothesis. Psychol Bull.

[57] Collinge W, Kahn J, Soltysik R (2012). Promoting reintegration of National Guard veterans and their partners using a self-directed program of integrative therapies: a pilot study. Mil Med.

[58] Gureje O, Von Korff M, Simon GE, Gater R (1998). Persistent pain and well-being: a World Health Organization Study in Primary Care. JAMA.

[59] Tian TY, Zlateva I, Anderson DR (2013). Using electronic health records data to identify patients with chronic pain in a primary care setting. J Am Med Inform Assoc.

[60] Cohen J (1988). Statistical Power Analysis For The Behavioral Sciences (2nd Edition).

[61] Strauss A, Corbin J (1998). Basics Of Qualitative Research: Second Edition: Techniques And Procedures For Developing Grounded Theory.

[62] Sandelowski M (1995). Sample size in qualitative research. Res Nurs Health.

[63] Ritenbaugh C, Nichter M, Nichter MA, Kelly KL, Sims CM, Bell IR, Castañeda HM, Elder CR, Koithan MS, Sutherland EG, Verhoef MJ, Warber SL, Coons SJ (2011). Developing a patient-centered outcome measure for complementary and alternative medicine therapies I: defining content and format. BMC Complement Altern Med.

[64] Thompson JJ, Kelly KL, Ritenbaugh C, Hopkins AL, Sims CM, Coons SJ (2011). Developing a patient-centered outcome measure for complementary and alternative medicine therapies II: refining content validity through cognitive interviews. BMC Complement Altern Med.

[65] Busija L, Pausenberger E, Haines TP, Haymes S, Buchbinder R, Osborne RH (2011). Adult measures of general health and health-related quality of life: Medical Outcomes Study Short Form 36-Item (SF-36) and Short Form 12-Item (SF-12) Health Surveys, Nottingham Health Profile (NHP), Sickness Impact Profile (SIP), Medical Outcomes Study Short Form 6D (SF-6D), Health Utilities Index Mark 3 (HUI3), Quality of Well-Being Scale (QWB), and Assessment of Quality of Life (AQoL). Arthritis Care Res (Hoboken).

[66] Polomano RC, Galloway KT, Kent ML, Brandon-Edwards H, Kwon KN, Morales C, Buckenmaier C (2016). Psychometric Testing of the Defense and Veterans Pain Rating Scale (DVPRS): A New Pain Scale for Military Population. Pain Med.

[67] Turk DC, Melzack R (1992). Self-report scales and procedures for assessing pain in adults. Handbook Of Pain Assessment, Third Edition.

[68] Clark ME, Gironda RJ, Young RW (2003). Development and validation of the Pain Outcomes Questionnaire-VA. J Rehabil Res Dev.

[69] Corrigan JD, Bogner J (2007). Initial reliability and validity of the Ohio State University TBI Identification Method. J Head Trauma Rehabil.

[70] Bovin MJ, Marx BP, Weathers FW, Gallagher MW, Rodriguez P, Schnurr PP, Keane TM (2016). Psychometric properties of the PTSD Checklist for Diagnostic and Statistical Manual of Mental Disorders-Fifth Edition (PCL-5) in veterans. Psychol Assess.

[71] Beck A T, Steer R A, Ball R, Ranieri W (1996). Comparison of Beck Depression Inventories -IA and -II in psychiatric outpatients. J Pers Assess.

[72] Cohen S, Kamarck T, Mermelstein R (1983). A global measure of perceived stress. J Health Soc Behav.

[73] Buysse DJ, Reynolds CF, Monk TH, Berman SR, Kupfer DJ (1989). The Pittsburgh Sleep Quality Index: a new instrument for psychiatric practice and research. Psychiatry Res.

[74] Busby DM, Christensen C, Crane DR, Larson JH (1995). Revision of the Dyadic Adjustment Scale for Use with Distressed Non distressed Couples: Construct Hierarchy Multidimensional Scales. J Marital Fam Ther.

[75] Neff KD (2003). The Development and Validation of a Scale to Measure Self-Compassion. Self and Identity.

[76] Sprecher S, Fehr B (2016). Compassionate love for close others and humanity. Journal of Social and Personal Relationships.

[77] Ten Have TR, Normand S-LT, Marcus SM, Brown CH, Lavori P, Duan N (2008). Intent-to-Treat vs. Non-Intent-to-Treat Analyses under Treatment Non-Adherence in Mental Health Randomized Trials. Psychiatr Ann.

[78] Kenny DA, Kashy DA, Cook WL (2019). Dyadic Data Analysis (methodology In The Social Sciences).

