# Supplementary material for: A Mobile and Web-Based Self-Directed Complementary and Integrative Health Program for Veterans and Their Partners (Mission Reconnect): Protocol for a Mixed-Methods Randomized Controlled Trial
Source: JMIR Res Protoc. 2019 May 13;8(5):e13666. doi: 10.2196/13666 (PMC6535978; doi:10.2196/13666)
Supplement: Multimedia Appendix 1 [file resprot_v8i5e13666_app1.pdf]

SUMMARY STATEMENT

PROGRAM CONTACT: **(Privileged Communication)** ***Release Date:*** **03/27/2018**

Revised Date:

Application Number: **1 I01 RX002775-01A1**

Principal Investigator

HAUN, JOLIE N.

Applicant Organization: JAMES A. HALEY VA MEDICAL CENTER

Review Group: **RRD4**
Psychological Health & Social Reintegration

Meeting Date: **02/27/2018** ***RFA/PA:*** **RX18-003**
Council: **MAY 2018**
Requested Start: **01/01/2019**

Project Title: **Delivering a Mobile and Web Based Self Directed Complementary And Integrative Health Program to Veterans and Their Partners to Manage Pain and PTSD**
SRG Action: **Impact Score:146 Percentile:16.5**

Human Subjects: **30-Human subjects involved - Certified, no SRG concerns**
Animal Subjects: **10-No live vertebrate animals involved for competing appl.**
Gender: **1A-Both genders, scientifically acceptable**
Minority: **1A-Minorities and non-minorities, scientifically acceptable**
Children: **1A-Both Children and Adults, scientifically acceptable**
Clinical Research - not NIH-defined Phase III Trial

Project Year	Direct Costs Requested
1	273,862
2	274,005
3	289,754
4	255,393
TOTAL	1,093,014

ADMINISTRATIVE BUDGET NOTE: The budget shown is the requested budget and has not been adjusted to reflect any recommendations made by reviewers. If an award is planned, the costs will be calculated by VA Office of Research and Development (ORD) staff based on the recommendations outlined in the BUDGET COMMENT section and any relevant ORD service-specific limitations.

SUMMARY OF DISCUSSION:
The Board met in Plenary Session and reviewed the above proposal considering all internal and external reviews. This document summarizes the major points of the discussion concerning the proposed project. In any further development of this project, the investigator should consider carefully all the issues reflected in this Summary of Discussion as well as the more detailed comments in the individual critiques.

GENERAL COMMENTS:
The board continues to be enthusiastic about this application and was pleased with the majority of revisions made based on previous critiques. The application was reviewed as significant and innovative with strong preliminary data and investigative team. The plan to track concurrent treatments was well received, however adding a medical chart review to determine whether treatment is evidence-based or not is needed. The letters of support helps to demonstrate that a pathway for Mission Reconnect could exist in VA, however, also incorporating some implementation-related work (e.g., adding relevant provider/clinical/administrative perspectives into the qualitative work proposed in Aim 3 to inform possible recommendations for implementation in the VA system) would further pave the way for future implementation. One reviewer continued to have concerns about this additional efficacy trial as designed and desired additional justification and rationale, including discussion of alternative designs and design decisions. Statistical concerns included random block sizes may be too large and clarification of whether sample size calculations are based on the longitudinal model. Plans to collect data on possible exposure to components of the treatment while in the wait-list control should be planned.

SUGGESTIONS:
See above.

COMMENTS ON THE BUDGET: None.

DESCRIPTION (provided by applicant):
Chronic pain is one of the most prevalent medical conditions in the Veteran population. Pain often presents with comorbid conditions, specifically post-traumatic stress disorder (PTSD). Comorbid chronic pain and PTSD significantly impact the quality of life of Veterans and their families. Multi-faceted therapies leveraging complementary and integrative health (CIH) are mandated within VA to complement clinical practice guidelines improve Veterans' quality of life and ability to function. This research will evaluate a CIH intervention to manage pain and PTSD related outcomes within a bio-psychosocial framework. The proposed intervention, Mission Reconnect (MR), a user-driven, dyadic, self-care management program delivered online and by mobile app that has previously shown to be effective in a non-clinically defined community-based Veteran/military population. This research is needed to test MR's effects in a clinically defined population as a complement to clinical services to assess for potential subsequent implementation within the Department of Veterans Affairs (VA). This proposal resubmission is responsive to Veterans' reported desire for CIH and several VA initiatives, including RR&D's current special areas of interest for non-pharmacological activity- based interventions for chronic pain impacting pain reduction, function and quality of life. The VA Secretary of Health Strategic Priorities and the emerging VA Whole Health Program identify access to CIH for pain and self-care management as a priority to achieve optimal Veteran health. To be responsive to these priorities the 2016 VA State-of-the-Art Conference (SOTA) and Comprehensive Addiction Recovery Act (CARA) mandated VA's commitment to conduct rigorous research to integrate non-pharmacological and CIH approaches into care, with emphasis on pain management. This proposal is also responsive to the VA's Opioid Safety Initiative (OSI) and Pain Care Mission which prioritize the need for nonpharmacological treatment options for pain. The short-term goal of this study is to determine the effects of MR on (1) chronic pain, PTSD and related outcomes and (2) relationship outcomes for Veterans and their partners. The long-term goal is to determine the effectiveness and sustainability of using CIH self-care management programs like MR to improve outcomes for Veterans with chronic pain

and PTSD, and their partners. We propose a four-year mixed-methods randomized controlled trial of MR with two arms (treatment & wait-list control) in a clinical sample of Veterans with comorbid pain and PTSD, and their partners (e.g., spouse). The specific aims are to: (Aim 1) Determine MR effectiveness for physical (pain, sleep), PTSD (intrusion, arousal, avoidance, numbing), and psychological (depression, stress, anxiety) symptoms, and global health (quality of life); (Aim 2) Determine MR effectiveness for social (relationship satisfaction, compassion for self/others) outcomes among Veterans and their partners; and (Aim 3) Describe Veteran and partner perceived value of MR in a sub-sample of participants. The sample will consist of Veteran and partner dyads (N = 228) at the Ann Arbor, Puget Sound, and Tampa VA facilities. Aim 1 & 2 data collection will include self-report assessment of 4-data points over a 4-month period to evaluate physical, psychological, and social outcomes. Eight weekly reports will also be collected for the first two months of MR use to assess MR utilization, and pain and stress levels. Aim 3 data collection will include telephone interviews from a randomly selected sub-sample of MR treatment group dyads (n = 42) to examine MR user experiences and their suggestions making MR useful for Veterans and their partners.

PUBLIC HEALTH RELEVANCE:
This project is responsive to RR&D's current special areas of interest for non-pharmacological activity-based interventions for chronic pain impacting pain reduction, function and quality of life. This project aligns with the VA mandate for complementary and integrative health (CIH) care for Veterans and their families. CIH complements traditional care for Veterans managing chronic conditions, such as chronic pain and PTSD. Mission Reconnect (MR) is a user-driven, dyadic, CIH self-care management program delivered remotely that teaches techniques the Veteran/partner dyad can use to reduce pain, anxiety and stress, promote well-being and improve relationship quality. The research goal is to evaluate MR as an approach to manage chronic pain and PTSD symptoms, for potential subsequent implementation. This study will possibly provide a model for establishing remote access and sustainable implementation of CIH within VA.

CRITIQUE 1

Significance and Innovation: The proposal is for a two-arm RCT comparing Mission Reconnect to a waitlist/ treatment as usual control in VA patients with confirmed pain and PTSD diagnoses. Mission Reconnect is a CIH program delivered via the web and mobile app that focuses on massage and mindfulness and includes Veteran's partners. Mission Reconnect has been previously developed and tested with NIH SBIR funding on a non-clinical community sample of Veterans. The current proposal extends this research by testing Mission Reconnect in a VA clinical sample. The proposal is strongly grounded within the biopsychosocial model. This project is significant because comorbid pain and PTSD is a common problem in VA patients and current interventions are often inadequate. The proposal is novel in several ways. First, it delivers the intervention via a website and mobile app allowing veterans who do not desire or are unable to engage in traditional in-person intervention to engage in care. Second, it involves the Veteran's partner which can increase engagement in care and help address common sources of relationship stress. Third, it is studying a highly accessible application of massage, which has received little past research attention. Overall, the background provides a good and clearly written rationale for this study. The revised proposal has been strengthened by providing additional background on types of partner-assisted interventions. Background now also includes a detailed review of the evidence for message and mindfulness. These additions indicate that the current proposal is building off existing evidence while investigating novel research questions.

Importance and Impact: This proposal addresses an important problem: co-morbid chronic pain and PTSD in VA patients. Should the intervention prove effective, it would provide a highly accessible intervention and a novel addition to tradition treatment.

Contribution to VHA: Should the aims of the project be achieved it could make an important contribution to VA health care services. The revised proposal now plans to directly address pain functioning and quality of life. This helps to further align the proposal with the mission of RR&D.

Methods (Data Analysis)**:** Overall, the proposal describes a well-designed two-arm RCT that includes 228 dyads in quantitative aims and a subsample of 42 dyads in the experimental group who will participate in qualitative interviews. The design has many strengths including the use of VA administrative data to identify their participant pool and a proactive method for participant recruitment. The measurement battery appears to assess all relevant domains and was designed to minimize participant burden. The plan for qualitative data collection, coding, and analysis is well developed and described. The primary analyses (GLMM), power calculations, and plan for missing data are all strong, as is the dissemination plan. The revised application clarified that the goal of this research is not to engage Veterans who are not currently engaged in care. This alleviated my concerns about the study targeting the wrong patient population. The revised application also addressed a previously noted concern that it would be difficult to detect the effect of Mission Reconnect in the midst of so many other treatments. Strategies to address this include carefully tracking concurrent treatment, stratifying randomization by treatment status and conducting subgroup analyses of the effect of MR in the presence and absence of evidence-based treatments. My only remaining concern is that the 2-items used to assess concurrent Pain and PTSD treatments are unlikely to be adequate. Patients may not be able to self-report whether the treatments they are receiving are evidenced-based treatment as defined by VA/ DOD Clinical Practices Guidelines. A medical chart review will be needed for this. I see that a CRPS chart review is conducted prior to randomization. A review for concurrent treatment could be added here. The revised application also more carefully defined chronic musculoskeletal pain.

Adequacy of Data: The proposed method is likely to be successful in collecting the proposed data. The proposal described a number of relevant preliminary studies that lend support to the investigative team's ability to conduct this study. Strengths of the preliminary data include consultation with a Veteran engagement board, and data from the NIH RCT on the clinical significance of declines in PTSD and pain among Veterans who received Mission Reconnect.

Project Organization and Management: A detailed project organization plan is included that describes how data collection will be coordinated across the three sites.

Investigator Qualifications: The investigative team, including the Principal Investigator (PI), is well qualified to conduct this research and all needed areas of expertise are covered by the Co-I's and consultants.

Facilities and Resources: Letters of support demonstrate good facility support and documented agreements with consultants. A new letter of support from the Office of Connected Care was added in the revised application. This letter states, "Connected Care does not typically engage with non-VA apps at the stage of evaluating effectiveness but if Mission Reconnect is determined to have positive outcomes for Veterans and their partners, OCC will engage in collaborative discussions with Dr. Haun's team to evaluate potential to support the Mission Reconnect commercial app for use in VHA healthcare facilities, for clinical use and for direct patient care." This alleviates concerns that there is no pathway for this app enter eventually become part of regular VA services. The scientific environment at all three sites is very good.

Human Subjects: No concerns were noted regarding risk to participants, potential benefits, knowledge to be gained, or data safety and monitoring.

Inclusion of Women, Minorities and Children: The proposal aims to recruit 25% female Veterans and expects that minorities will be well-represented. No children will be included.

Budget (unscored)**:** The budget consists of salary for project staff, a few Co-Is and a statistical consultant. Money is also included for participant reimbursement, two trips for the PI, transcription services, and Qualtrics data collection. The revised budget justification provides full detail on why 190 hours of consultation services are needed and what is included in the $40k for “Web-infrastructure Program Management”. I have no concerns about any of these costs.

Data Management and Access Plan (for data sharing, unscored)**:** Adequate.

Overall Strengths:
- Proposal targets important problems in VA.
- Proposal is innovative in its use of a technology platform, the inclusion of Veteran’s partners, and the study of an accessible application of massage therapy.
- Strong preliminary data, especially from the NIH study, support the rationale to conduct this study in a VA clinical sample.
- Strategies for recruitment, assessment, and patient retention are strong.
- Strong investigative team.
- Strong support from VACO partners.

Overall Weaknesses: All major weaknesses have been addressed in the revisions. The investigators should make sure that CPRS is being used to code pain and PTSD services as evidence-based or not evidence- based.

CRITIQUE 2

Significance and Innovation: The revised proposal addressed this reviewer’s prior critiques in this area. The proposal now addresses the family studies literature, including typology of family/couple interventions, and the alignment of Mission Reconnect with partner/family-assisted interventions. Further justification has been added regarding the need for another RCT/efficacy trial for Mission Reconnect. This reviewer does not find the justification provided, however, completely satisfactory or sufficient. What about other designs such as hybrid designs?

Importance and Impact: As noted before, a strong case is made for the importance of the problem that the proposed research is seeking to address (i.e., comorbid chronic pain and PTSD) and how solving this problem would affect health care delivery and outcomes in VA, nationwide, and beyond.

Contribution to VHA: Again, as noted before, this project has the potential to greatly contribute to VHA and is clearly aligned with the mission of the Rehabilitation Research and Development (RR&D) Service. Letters indicating operational support are a strength (e.g., letter of support provided by the Executive Director of the Office of Centered Care and Cultural Transformation [OPCCT], which leads the Whole Health Program). As mentioned in the proposal, “this proposal resubmission is responsive to RR&D’s identified areas of interest this round in non-pharmacological activity-based interventions for chronic pain”.

Methods (Data Analysis)**:** The points I previously made below have been addressed:
- Please expand and clarify the rationale for the timing and number of assessments.
- The analysis plan does not appear to address the weekly reports.
- It is not clear whether the telephone interviews proposed as part of Aim 3 will be conducted separately for Veterans and partners or jointly as a dyad, which has important methodological implications.
- Relationship satisfaction is a secondary outcome. Is there concern with ceiling effects at baseline which have been seen in other studies?

Adequacy of Data: As before, Data Management and Access Plan is reasonable. Preliminary studies section is strong, including the prior study on Mission Reconnect and the pilot study conducted specifically for proposal submission on Veteran use of CIH and readiness for Mission Reconnect.

Project Organization and Management: As noted before, the study timeline provided seems reasonable. Sufficient time is allocated for data preparation, management, and analysis, which is often overlooked/underestimated. There is a detailed plan for project organization and management, which is necessary and helpful in this case given the number of sites and individuals involved. The number of sites and individuals involved does add a level of complexity with a number of opportunities for miscommunication, etc.

Investigator Qualifications: See my prior review comments in this area. The revised proposal is very responsive. Dr. Shirley Glynn is a great addition to the team. This reviewer had noted that additional expertise in family studies may be warranted. A table has been added to highlight the complementary and integrated nature of the team. The revised proposal also addresses that the PI has not previously led a large clinical trial.

Facilities and Resources: As noted before, facilitates and resources are sufficient. The fact that the Veteran Engagement Panel reviewed the proposal is a strength. The revised proposal now includes details on feedback received and changes/decisions made as a result.

Human Subjects: No concerns related to proposed use of human participants and protections from research risk relation to their participation. Risk to participants; adequacy of protection against risks; potential benefits of the proposed research to the participants and others; importance of the knowledge to be gained; and data and safety monitoring were all addressed.

Vertebrate Animals: Not Applicable

Biohazard and Radioisotopes: Not Applicable.

Inclusion of Women, Minorities and Children: The revised proposal explains how women and minorities recruitment will mirror site distribution based on sex and race. The proposal now addresses how this distribution will be achieved and strategies/plans for any difficulties in reaching these numbers.

Budget (unscored)**:** No concerns. Detail has been added to the budget justification for Mission Reconnect, LLC expenses. Documentation has been provided that Qualtrics is approved for this particular project, etc.

Data Management and Access Plan (for data sharing, unscored)**:** No concerns. DMAP has been revised to address qualitative data. What does a de-identified qualitative data set contain/look like?

Overall Strengths:
- A strong case is made for the importance of the problem that the proposed research is seeking to address (i.e., comorbid chronic pain and PTSD) and the impact of solving this problem.
- Potential to contribute to VHA and complement existing services/programs (operational support provided). Clear alignment with mission of RR&D.
- Preliminary studies and pilot data. Also, use of biopsychosocial model and conceptual framework to guide the proposed study.
- Very responsive resubmission.

Overall Weaknesses: Rationale for RCT/efficacy trial still not sufficient/satisfactory. More could also be done to ensure/inform pathway to incorporate Mission Reconnect into regular clinical care in the future (e.g., implementation-related aim).

CRITIQUE 3

Significance and Innovation: No comment.

Importance and Impact: No comment.

Contribution to VHA: No comment.

Methods (Data Analysis)**:**
- This is a well-designed study with a few minor points.
  - Given the overall size of the study, random block sizes of 6 and 8 maybe too large and could lead to imbalances in the sample size. Smaller block sizes (2 and 4) would minimize the maximum size of an imbalance.
  - The investigators clearly justify the use of a wait-list control. A concern is that participants assigned to the control condition may, on their own, seek care such as massage. While the investigators cannot prevent this, they should measure it by collecting information on the interventions that have been used while on the wait list.
  - The analysis plan seems to be appropriate but it is not clear how the plan maps to the sample size calculations. It is not clear if the sample size calculations are based on the longitudinal model that is outlined in the analytic plan.

Adequacy of Data: Include assessment of MR utilization and an assessment of the interventions undertaken while on the wait list.

Project Organization and Management: No comment.

Investigator Qualifications: No comment.

Facilities and Resources: No comment.

Human Subjects: A data monitoring plan is included in the Human Subject section and is appropriate. One thing to consider is how to maintain the masking of the investigators while conducting the monitoring.

Vertebrate Animals: Not Applicable.

Biohazard and Radioisotopes: Not Applicable.

Inclusion of Women, Minorities and Children: Appropriate.

Budget (unscored)**:** Appropriate.

Data Management and Access Plan (for data sharing, unscored)**:**
- Data management is appropriate.
- The investigators indicate that they will share data.

Overall Strengths: No comment.

Overall Weaknesses: No comment.

MEETING ROSTER

Psychological Health & Social Reintegration
Rehabilitation Research and Development Parent IRG
Office of Research & Development
RRD4
02/27/2018

CHAIRPERSON(S)

HORAN, WILLIAM P., PHD
ASSOCIATE RESEARCH PSYCHOLOGIST
VA GREATER LOS ANGELES HEALTHCARE SYSTEM
DEPT OF PSYCHIATRY AND BEHAVIORAL SCIENCES
UNIVERSITY OF CALIFORNIA, LOS ANGELES
LOS ANGELES, CA 90073

HUNT, PETER CODY, PHD
REHABILITATION SCIENTIST,
SPINAL CORD INJURY/DISORDERS HEALTH CARE GROUP
VA LONG BEACH HEALTHCARE SYSTEM
LONG BEACH, CA 90822

MEMBERS

ACION, LAURA, PHD *
ASSOCIATE RESEARCH SCIENTIST
IOWA CONSORTIUM FOR SUBSTANCE ABUSE
RESEARCH AND EVALUATION
UNIVERSITY OF IOWA
IOWA CITY, IA 52242

BEALS, KIM PHD *
ASSISTANT PROFESSOR
DEPARTMENT OF SPORTS MEDICINE AND NUTRITION
SCHOOL OF HEALTH & REHABILITATION SCIENCES
UNIVERSITY OF PITTSBURGH
PITTSBURGH, PA 15203

BELANGER, STEPHANIE, PHD *
CO SCIENTIFIC DIRECTOR (INTERIM)
CANADIAN INSTITUTE FOR MILITARY AND
VETERAN HEALTH RESEARCH
ASSOCIATE PROFESSOR
ROYAL MILITARY COLLEGE OF CANADA
KINGSTON, ON K7K 7B4

BERTOCCI, KAREN LEIGH *
ASSOCIATE PROFESSOR
UNIVERSITY OF LOUISVILLE
500 SOUTH PRESTON STREE
LOUISEVILLE, KY 40202

BHAUMIK, DULAL KUMAR, PHD *
PROFESSOR
EPIDEMIOLOGY & BIOSTATISTICS
CENTER FOR HEALTH STATISTICS
UNIVERSITY OF ILLINOIS, CHICAGO
CHICAGO, IL 60612

BOWEN, MARY E., PHD *
RESEARCH HEALTH SCIENCE SPECIALIST
PHILADELPHIA VA MEDICAL CENTER
ASSOCIATE PROF, WEST CHESTER UNIVERSITY OF PENN
UNIVERSITY OF PENN SCHOOL OF NURSING
PHILADELPHIA, PA 19104

COPELAND, LAUREL A., PHD
ASSOCIATE DIRECTOR
CENTER FOR APPLIED HEALTH RESEARCH
CENTRAL TEXAS VETERANS HEALTH CARE SYSTEM
TEMPLE, TX 76502

DAVIS, LOUANNE W., PSYD *
CLINICAL RESEARCH PSYCHOLOGIST
RICHARD L. ROUDEBUSH VA MEDICAL CENTER
ASSOCIATE SCIENTIST IN CLINICAL PSYCHOLOGY
INDIANA UNIVERSITY SCHOOL OF MEDICINE
INDIANAPOLIS, IN 46202

DRAPALSKI, AMY L, PHD *
CLINICAL RESEARCH PSYCHOLOGIST
VISN 5 MENTAL ILLNESS RSEARCH
EDUCATION AND CLINICAL CENTER
VA MARYLAND HEALTHCARE SYSTEM
BALTIMORE, MD 21201

GREENBERG, BENJAMIN D, PHD, MD *
ASSOCIATE DIRECTOR
CENTER FOR NEURORESTORATION & NEUROTECHNOLOGY
PROVIDENCE VA MEDICAL CENTER
PROFESSOR - PSYCHIATRY & HUMAN BEHAVIOR
BROWN UNIVERSITY - BUTLER HOSPITAL
PROVIDENCE, RI 02906

GRIFFITHS, PATRICIA C., PHD *
RESEARCH SCIENTIST
ASSIST DIRECTOR FOR OUTREACH AND EDUC
ATLANTA VA MEDICAL CENTER RR&D CVNR
ASSIST PROFESSOR, DIV OF GEN MED AND GERIATRICS
EMORY UNIVERSITY
DECATUR, GA 30033

HEAPY, ALICIA, PHD *
ASSOCIATE DIRECTOR
RESEARCH ENHANCEMENT AWARD PROGRAM
VA CONNECTICUT HEALTH CARE SYSTEM - WEST HAVEN
ASSISTANT PROFESSOR PSYCHOLOGY
YALE SCHOOL OF MEDICINE
WEST HAVEN, CT 06516

KING, HEATHER A, PHD *
RESEARCH HEALTH SCIENTIST
DURHAM VA MEDICAL CENTER
HEALTH SERVICES RESEARCH AND DEVELOPMENT
ASSISTANT PROFESSOR, DEPARTMENT OF MEDICINE
DUKE MEDICINE
DURHAM, NC 27705

KING, PAUL R. JR, PHD
CLINICAL RESEARCH PSYCHOLOGIST AND ASSOCIATE
DIRECTOR OF POSTDOCTORAL TRAINING
VISN 2 CENTER FOR INTEGRATED HEALTHCARE
ADJUNCT ASSISTANT PROFESSOR
DEPT OF COUNSELING, SCHOOL, & EDUCATIONAL PSYCH
UNIVERSITY OF BUFFALO
BUFFALO, NY 14215

MCDONALD, SCOTT D., PHD *
CO-DIRECTOR
RICHMOND VA MEDICAL CENTER
ASSISTANT PROFESSOR
DEPTS OF PHYSICAL MED & REHAB AND PSYCHOLOGY
VIRGINIA COMMONWEALTH UNIVERSITY
RICHMOND, VA 23249

NUGENT, ALLISON C., PHD
STAFF SCIENTIST
DIRECTOR OF NEUROIMAGING RESEARCH, EXPERIMENTAL
THERAPEUTICS AND PATHOPHYSIOLOGY BRAND
NATIONAL INSTITUTE ON MENTAL HEALTH
DEPT OF HEALTH AND HUMAN SERVICES
BETHESDA, MD 20892

ORR, JOSEPH M., PHD *
ASSISTANT PROFESSOR
DEPARTMENT OF PSYCHOLOGY
TEXAS A&M UNIVERSITY
COLLEGE STATION, TX 77843

POSSEMATO, KYLE, PHD *
CLINICAL RESEARCH PSYCHOLOGIST
ASSOCIATE DIRECTOR FOR RESEARCH
CENTER FOR INTEGRATED HEALTHCARE
SYRACUSE VA MEDICAL CENTER
SYRACUSE, NY 13210

SUGAR, CATHERINE ANN, PHD *
ASSOCIATE PROFESSOR
DEPARTMENT OF BIOSTATISTICS
UCLA SCHOOL OF PUBLIC HEALTH
PSYCHIATRY AND BIOBEHAVIORAL SCIENCES
GEFFEN SCHOOL OF MEDICINE
LOS ANGELES, CA 90095

THURAS, PAUL D., PHD *
RESEARCH AND EVALUATION PROGRAM DIRECTOR
MENTAL HEALTH PSL
MINNEAPOLIS VA MEDICAL CENTER
MINNEAPOLIS, MN 55417

WEISENBACH, SARA L., PHD *
DEPARTMENT OF PSYCHIATRY
UNIVERISTY OF UTAH
SALT LAKE CITY, UT 84112

WISNIEWSKI, STEPHEN R, PHD *
PROFESSOR
DEPARTMENT OF EPIDEMIOLOGY
EPIDEMIOLOGY DATA CENTER
UNIVERSITY OF PITTSBURGH
PITTSBURGH, PA 15260

YOON, JONG H, MD *
PHYSICIAN
PALO ALTO VA MEDICAL CENTER
ASSISTANT PROFESSOR
PSYCHIATRY AND BEHAVIORAL SCIENCES
STANFORD UNIVERSITY
STANFORD, CA 94305

EXECUTIVE SECRETARY

BOUWER, H. G. ARCHIE, PHD
DEPUTY CHIEF OF STAFF
VA PORTLAND HEALTH CARE SYSTEM
SOM-MOLECULAR MICROBIOLOGY & IMMUNOLOGY DEPT
OREGON HEALTH SCIENCES UNIVERSITY
PORTLAND, OR 97213

SCIENTIFIC REVIEW OFFICER

GROER, SHIRLEY, PHD
SCIENTIFIC PROGRAM MANAGER
DEPARTMENT OF VETERANS AFFAIRS
OFFICE OF RESEARCH AND DEVELOPMENT
REHABILITATION RESEARCH AND DEVELOPMENT SERVICE
WASHINGTON, DC 20420

* Temporary Member. For grant applications, temporary members may participate in the entire meeting or may review only selected applications as needed.

Consultants are required to absent themselves from the room during the review of any application if their presence would constitute or appear to constitute a conflict of interest.
